# Ecologically Different Fungi Affect *Arabidopsis* Development: Contribution of Soluble and Volatile Compounds

**DOI:** 10.1371/journal.pone.0168236

**Published:** 2016-12-14

**Authors:** Salvatore Casarrubia, Sara Sapienza, Héma Fritz, Stefania Daghino, Maaria Rosenkranz, Jörg-Peter Schnitzler, Francis Martin, Silvia Perotto, Elena Martino

**Affiliations:** 1 Department of Life Sciences and Systems Biology, University of Turin, Turin, Italy; 2 INRA-Nancy and Lorraine University, Lab of Excellence ARBRE, Unité Mixte de Recherche 1136, Champenoux, France; 3 Research Unit Environmental Simulation, Institute of Biochemical Plant Pathology, Helmholtz Zentrum München—German Research Center for Environmental Health (GmbH), Neuherberg, Germany; Estacion Experimental del Zaidin, SPAIN

## Abstract

Plant growth and development can be influenced by mutualistic and non-mutualistic microorganisms. We investigated the ability of the ericoid endomycorrhizal fungus *Oidiodendron maius* to influence growth and development of the non-host plant *Arabidopsis thaliana*. Different experimental setups (non-compartmented and compartmented co-culture plates) were used to investigate the influence of both soluble and volatile fungal molecules on the plant phenotype. *O*. *maius* promoted growth of *A*. *thaliana* in all experimental setups. In addition, a peculiar clumped root phenotype, characterized by shortening of the primary root and by an increase of lateral root length and number, was observed in *A*. *thaliana* only in the non-compartmented plates, suggesting that soluble diffusible molecules are responsible for this root morphology. Fungal auxin does not seem to be involved in plant growth promotion and in the clumped root phenotype because co-cultivation with *O*. *maius* did not change auxin accumulation in plant tissues, as assessed in plants carrying the DR5::GUS reporter construct. In addition, no correlation between the amount of fungal auxin produced and the plant root phenotype was observed in an *O*. *maius* mutant unable to induce the clumped root phenotype in *A*. *thaliana*. Addition of active charcoal, a VOC absorbant, in the compartmented plates did not modify plant growth promotion, suggesting that VOCs are not involved in this phenomenon. The low VOCs emission measured for *O*. *maius* further corroborated this hypothesis. By contrast, the addition of CO_2_ traps in the compartmented plates drastically reduced plant growth, suggesting involvement of fungal CO_2_ in plant growth promotion. Other mycorrhizal fungi, as well as a saprotrophic and a pathogenic fungus, were also tested with the same experimental setups. In the non-compartmented plates, most fungi promoted *A*. *thaliana* growth and some could induce the clumped root phenotype. In the compartmented plate experiments, a general induction of plant growth was observed for most other fungi, especially those producing higher biomass, further strengthening the role of a nonspecific mechanism, such as CO_2_ emission.

## Introduction

Plant-associated microorganisms are essential drivers of plant productivity because they can increase nutrient availability and uptake, enhance stress tolerance, provide disease resistance and expand plant metabolic pathways [1,2]. They also play important functions in plant development, and functional traits such as leaf development, shoot/root ratio and root architecture may undergo substantial changes following plant-microorganism interactions [2,3]. In particular, plant growth-promoting rhizobacteria (PGPR; [4]) and rhizospheric fungi (PGPF [5]) are able to promote plant growth and development thanks to direct and indirect mechanisms. Indirect mechanisms include improved mineral nutrition through mineral solubilisation or disease suppression, whereas direct mechanisms involve production of phytohormones and volatile organic compounds (VOCs) [6]. Many PGPR strains can release volatile mixtures that stimulate plant growth [7].

Plant growth and development may be influenced by the interaction with either beneficial or pathogenic microorganisms [3,6]. For example, several non-mutualistic PGPR modify plant root architecture by increasing primary root length, lateral root number, length and density, or root hair formation [8–12]. Several non-mutualistic fungi and root-associated endophytic fungi have been described in the literature as PGPF. Among them are strains belonging to the genera *Penicillium*, *Fusarium*, *Phoma*, *Trichoderma*, *Ampelomyces*, *Coniothyrium*, *Aspergillus*, *Sarocladium*, *Ophiosphaerella*, *Piriformospora* [6,13–19]. The mycorrhizal symbiosis facilitates water and nutrient absorption and positively affects plant growth [20]. For example, roots colonized by arbuscular mycorrhizal (AM) fungi display enhanced root biomass and increased lateral roots if compared to non-AM roots [21–24]. Ectomycorrhizal (ECM) fungi also increase root growth and lateral root formation in their host plants, which typically display numerous short lateral roots [25–30]. Several studies have shown stimulation of lateral root development in the host plant in the very early phase of the ECM interaction, prior to symbiosis establishment, suggesting that soluble diffusible/volatile signalling molecules are responsible for changes in root architecture in this early phase [29,31,32]. Some ECM fungi can also induce lateral root development in the non-host plant *Arabidopsis thaliana*, indicating that signalling is non-host specific and that the root phenotype does not depend on the plant’s ability to form ECM [29,31,32]. Felten et al. [29] and Splivallo et al. [31] demonstrated that fungal-derived indole-3-acetic acid (IAA) plays an important role in modifying root morphology, and that fungal-derived ethylene may influence plant lateral root development and branching in the early stages of interaction. ECM fungi may thus modify the endogenous hormonal plant balance, and Felten et al. [29] proposed a model where the fungus induces auxin accumulation at the root apex, thus stimulating lateral root formation. In addition to soluble phytohormones, fungi also emit species-specific blends of VOCs. The emission intensity as well as the VOCs composition depends strongly on physiological as well as environmental factors [33,34]. Recently, it was shown that plants could sense fungal emitted sesquiterpenes [32,35]; the airborne plant-fungal communication through the sesquiterpene thujopsene was suggested to prepare the plants for mycorrhizal symbiosis [32].

Ericoid mycorrhizal (ERM) fungi are soil-born fungi mostly belonging to Leotiomycetes (Ascomycetes) and form symbiotic associations with plants in the family Ericaceae [36], in which they induce plant growth under stressful conditions [37,38]. The influence of ERM fungi on the growth of other plant species has never been investigated, despite the identification of ERM-related fungi in association with the roots of non-ericaceous plants [39]. We have investigated the influence of the ERM fungus *Oidiodendron maius* [40] on growth and development of the non-mycorrhizal model plant *A*. *thaliana*. We used different experimental setups to investigate the role of both soluble and volatile molecules on the plant phenotype, and compared *O*. *maius* with nine other fungi comprising diverse ecological strategies, as well as with some *O*. *maius* mutants. *O*. *maius* strongly promoted plant growth in *A*. *thaliana* and induced a peculiar root phenotype that was also induced by other fungal species, but not by an *O*. *maius* mutant with altered nitrogen pathways. Unlike ECM fungi, *O*. *maius* does not seem to resort on IAA or VOCs to promote growth. Instead, the results underlined a major non-specific contribution of fungal emitted CO_2_ to plant growth promotion in our *in vitro* experimental setup.

## Materials and Methods

### Fungal strains and culture media

*Oidiodendron maius* strain Zn is deposited at the *Mycotheca Universitatis Taurinensis* collection (MUT1381) of the Department of Life Sciences and Systems Biology (University of Turin, Italy) and at the American Type Culture Collection (ATCC MYA-4765) of the University of Boulevard (Manassas, VA, US). This strain was isolated from the Niepolomice Forest (25 km northeast of Krakow, Poland) from the roots of *Vaccinium myrtillus* plants growing in experimental plots treated with dust containing high concentrations of heavy metals [41]. Nine fungal strains, obtained from the INRA fungal collection (Nancy, France), were used for comparison. They include three other ERM strains (*Meliniomyces bicolor*, *M*. *variabilis* and *Rhizoscyphus ericae*), three ECM strains (*Cenococcum geophilum*, *Laccaria bicolor* strain S238N and *Suillus luteus*), one orchid mycorrhizal strain (*Tulasnella calospora*), one white rot saprothrophic fungus (*Trametes versicolor*) and one pathogenic fungus (*Cladosporium herbarum*). Three characterized *O*. *maius* mutants were also used for comparative experiments: the *O*. *maius* SOD mutant [42], the *O*. *maius* mutant carrying a disruption on a gene belonging to the Major Facilitator Superfamily (MFS) transporter family (Abbà, unpublished) and the *O*. *maius* GOGAT mutant [43]. This last mutant carries a partial deletion of the glutamate synthase (NADH-GOGAT) gene [43]. The OmGOGAT disruption modifies the nitrogen pathway and this mutant has an altered nitrogen metabolism. All fungal strains were maintained on Czapek-glucose solid medium (NaNO_3_ 3 g/L, K_2_HPO_4_*3H_2_O 1.31 g/L, MgSO_4_*7H_2_O 0.5 g/L, FeSO4*7H_2_O 0.01 g/L, KCl 0.5 g/L, glucose 20 g/L, agar 10 g/L). The medium was adjusted to pH 6 with the addition of 1 M HCl. All reagents were purchased from SIGMA.

### Plant growth

Seeds of *A*. *thaliana* (the ecotype Col-0 and DR5::GUS transformant) were surface sterilized with a solution containing 70% ethanol for 10 minutes and 100% ethanol for few seconds. Then seeds were dried 2–3 h in the sterile hood and transferred on 1% agar medium containing 2.29 g/L half strength MS [44] medium (Murashige and Skoog Basal Salts Mixture including vitamins), 10 g/L sucrose, 1 g/L MES (2-N-morpholino ethane sulphonic acid) sodium salt (pH 5.8–6) for germination. Plates were kept at 4°C for 2 days and then they were placed vertically in a plant growth chamber (16-h photoperiod, light at 170 μmol m^–2^s^–1^, temperature at 23°C day and 21°C night) for 5–7 days.

### Cellophane and cellulose nitrate membrane preparation

When necessary, prior to inoculation, sterile cellophane or cellulose nitrate membranes were placed aseptically on the agar surface to provide a convenient means of removing/transferring mycelia or plants. Cellophane membranes were prepared by first boiling for 30 min in 10 mM EDTA (disodium salt, dihydrate, SIGMA), rinsing and then autoclaving in ddH_2_O, while cellulose nitrate membranes were autoclaved and then dried over night at 50°C.

### Co-culturing of fungi and *A*. *thaliana* plants

Co-culturing of fungi with *A*. *thaliana* plants included three different experimental setups. **(1) Non-compartmented square plates.** Twelve centimeters square petri dishes containing MS medium were used and five *A*. *thaliana* germinated seedlings prepared as described above were placed on the half-upper side of each petri dish. When the *A*. *thaliana* seedlings’ roots length reached 5 cm, the half-bottom side of the petri dish was inoculated with fungi. Two different systems were used for fungal inoculation: (a) three 0.5-cm mycelial plugs were placed directly on the MS solidified medium; (b) three 0.5-cm mycelial plugs of *O*. *maius* were previously grown for 15 days on cellulose nitrate membranes (Scheicher&Schuell, ME24ST, 0.2 μm, 47 mm) in MS containing petri plates. The membranes covered by the mycelium were then placed fungus side down (direct interaction) or fungus side up (indirect interaction) on the *A*. *thaliana* roots. Inoculated plates (including non-inoculated control plates) were placed vertically in a growth chamber (16-h photoperiod, light at 170 μmol m^–2^ s^–1^, temperature at 23°C day and 21°C night) for 30 days. **(2) Bi-partite plates.** Nine centimeters round bi-partite petri dishes containing MS medium were used. Three *A*. *thaliana* seedlings, prepared as described above, were placed in the upper part of the right plate compartment, while one fungal plug was inoculated in the middle of the left plate compartment previously covered with a sterile cellophane membrane prepared as described above. Control plates without fungi were also prepared. In this setup, the mycelial exudates do not diffuse to the plant compartment and only fungal volatiles can reach the seedlings. These plates were placed vertically in a growth chamber (16-h photoperiod, light at 170 μmol m^–2^ s^–1^, temperature at 23°C day and 21°C night) for two weeks. **(3) Tri-partite plates with volatile and CO**_**2**_ **traps.** Round nine centimeters tri-partite petri dishes were used. Three *A*. *thaliana* seedlings, prepared as described above, were placed in one compartment and one 0.5 cm fungal plug was inoculated in the second compartment previously covered with a sterile cellophane membrane prepared as described above. Both compartments contained MS medium. The third compartment was filled with volatile or CO_2_ traps. The volatile trap was made of 2 g of activated charcoal (untreated granular 8–20 mesh—C2889 Sigma-Aldrich). As CO_2_ trap, 7 ml of 0.1 M Ba(OH)_2*_8H_2_0 were added together with two dental rolls to avoid barium hydroxide spillage in the neighboring plate compartments. 7 ml of 1 M Ca(OH)_2_ and 0.9 g of Ca(OH)_2_ (solid form) were also tested as CO_2_ traps. Control plates without fungi, control plates without the two trap compounds and control plates with a CO_2_ saturated barium hydroxide solution were also prepared. These plates were placed horizontally in a growth chamber (16-h photoperiod, light at 170 μmol m^–2^ s^–1^, temperature at 23°C day and 21°C night) for two weeks.

Five plates, each containing three to five plants, were prepared and analyzed for each treatment. Fresh and dry weights of mycelia, roots and aboveground (stem + leaves) plant portions from all the different co-culture systems described were determined. After harvest, mycelia and plants were blotted dry on a paper towel to remove agar and water excess, and fresh weights (FW) were measured. Plant dry weights (DW) were measured after drying plant material in a ventilated oven at 60°C to a constant weight. Plant images were recorded by using a Nikon eclipse E300 system.

### Morphological analysis of *A*. *thaliana* root development

Twenty individual *A*. *thaliana* plants for each condition (five plants per Petri plate, 4 replicates) were observed every three days up to 12 days in a control experiment and after co-cultivation with *O*. *maius* WT or with the *O*. *maius* GOGAT mutant. For the quantification of root parameters, plants after 9 days of plant-fungus co-cultivation were considered because modifications of the root phenotype were already visible at this developmental stage, and roots had not touched the plate side either in the control plates or in the co-cultivation plates. Images of the whole plants were acquired with an Epson Perfection V300 scanner (Epson America, USA) at 600 dpi and saved in TIFF format. Primary and lateral roots (LRs) were counted and measured using the ImageJ plug in SmartRoot software [45].

### Sampling and analysis of fungal VOCs

*O*. *maius* was grown at 25°C in plastic (6 replicates) and glass (3 replicates) petri plates containing MS medium covered with cellophane membranes. VOCs were collected in the cultures headspace after 15 and 30 days from fungal inoculum. Control plates without mycelium were sampled for background correction. VOCs were collected for 6 h from sealed Petri dishes by headspace sorptive extraction using the stir bar sorptive extraction method with Gerstel Twisters (Gerstel GmbH & Co. KG, Mülheim an der Ruhr, Germany) as described in [33]. The samples were analysed with a thermo-desorption unit (Gerstel GmbH & Co) coupled to a gas chromatograph-mass spectrometer (GC-MS; GC model: 7890A; MS model: 5975C; Agilent Technologies, Santa Clara, CA, USA) as described in [34]. The chromatograms were analyzed by the enhanced ChemStation software (MSD ChemStation E.02.01.1177, 1989–2010 Agilent Technologies, Santa Clara, CA, USA). The TIC (Total Inorganic Carbon) of each VOC in the final dataset was recalculated from the absolute abundance of the first representative m/z to eliminate noise. The calibration was done as described in [34]. The emission rates were calculated on fungal mycelium area (pmol cm^-2^ h^-1^) bases.

### GUS assay

The GUS assay was done on aboveground (stems, leaves) and belowground portions of *A*. *thaliana* fresh tissues, sampled after 7 days of co-cultivation in non-compartmented plates. Each sample was incubated with the GUS-buffer (0.1 M sodium phosphate buffer pH 7; 5 mM K_4_Fe(CN)_6_; 5 mM K_3_Fe(CN)_6_), 0.1% Triton X100, 0.1% x-GlcA (5-Bromo-4-chloro-3-indolyl-ß-D-glucuronic acid) reagent (Duchefa Biochemie), 1 mM EDTA for 16h at 37°C in the dark and then washed with 70% ethanol. Tissues were observed and photographed using a Nikon Eclipse E400 optical microscope.

### Auxin measurement using Salkowski reaction

Two mycelial plugs of *O*. *maius* WT, *O*. *maius* GOGAT mutant, *O*. *maius* MFS mutant and *O*. *maius* SOD mutant were used to inoculate three flasks each containing 40 ml of MS liquid medium. Three flasks containing the growth medium were used as a negative control. Flasks were kept on a shaking incubator at 120 rpm at 25°C in the dark. After twenty days of culture, fungal growing media were vacuum filtered through filter paper disks and concentrated through lyophilization. Three 1 ml aliquots of 8X concentrated samples were prepared and a colorimetric assay was used to estimate the concentration of indole compounds by mixing the supernatant with 1 ml of Salkowski reagent (0.138 M FeCl_3_ and 7.9 M H_2_SO_4_) [46]. Samples were incubated at room temperature for 30 min in the dark and analyzed at 530 nm on a spectrophotometer (Beckman DU^®^530). IAA levels were determined with an IAA standard curve using commercial IAA (Duchefa—Biochemie) and sterile medium as a blank. IAA levels were expressed as μg/g of dry mycelium.

### Statistical analysis

The significance of differences among the different treatments was statistically evaluated by ANOVA with Tukey’s pairwise comparison as post hoc test for multiple comparisons for normal distributed data. Kruskall-Wallis with Bonferroni-corrected pairwise Mann-Whitney post-hoc adjustment were used as statistical test for non-normal distributed data. Pearson’s correlation test was performed to measure the strength of the association between plant and fungal biomass values obtained in the bi-partite plate assay, considering for the significance of the correlation a probability level <0.01. Statistical elaborations of growth and biomass data were performed using PAST statistical package, version 2.17 [47]. For statistical analysis of the VOC samples, Kruskall-Wallis test with Dunnet T3 post hoc test was used. For dependent samples Wilcoxons test was used. The differences were considered significant at a probability level of P<0.05.

## Results

### *O*. *maius* positively influences development of the non-host plant *A*. *thaliana*

**Fig 1:**
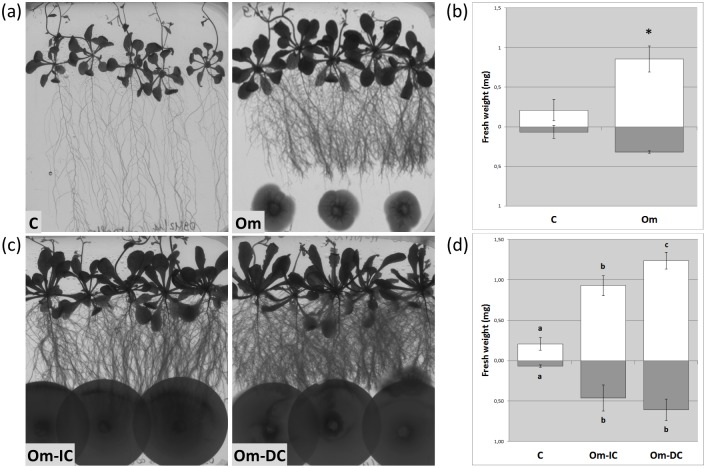
*A*. *thaliana* development in the presence of *O*. *maius*. (a) *A*. *thaliana* control plants (C) and *A*. *thaliana*-*O*. *maius* co-cultures (Om) 30 days after inoculation; (b) plant biomass measurements (roots—grey bars—and aboveground portions—open bars) in the presence (Om)/absence (C) of *O*. *maius*. Note the strong plant biomass increase in the presence of the fungus; (c) *A*. *thaliana*-*O*. *maius* co-cultures 30 days after inoculation: the fungus was previously grown on cellulose nitrate disks which were then placed on *A*. *thaliana* roots fungus side up (indirect contact—Om-IC) or fungus side down (direct contact—Om-DC); (d) plant biomass measurements (roots—grey bars—and aboveground portions—open bars) in the presence/absence of *O*. *maius* in indirect/direct contact with plant roots. Note the strong plant biomass increase in the presence of the fungus in both conditions and especially in the direct contact one. All pictures were taken at the same magnification. Bars represent the mean ±SD, n = 5 (each biological replicate represents the total biomass of 5 *A*. *thaliana* seedlings grown in an individual plate). Statistically significant differences (P<0.05) among treatments are indicated by asterisks or by different letters above the bars.

When co-cultured with *O*. *maius* in Petri dishes, the biomass of both roots and aerial parts of *A*. *thaliana* significantly increased (Fig 1). In particular, a 4-fold biomass increase of the aerial parts and a 5-fold increase of the root biomass were measured after direct inoculation of *O*. *maius* on the solid medium (Fig 1a and 1b). If the fungus was inoculated on a cellulose nitrate membrane before its transfer to the co-culture plates, a significant biomass increase was observed in *A*. *thaliana* irrespective of the membrane side in contact with the roots (Fig 1c and 1d). In this last experiment, a significantly (P<0.05) higher biomass of the aerial portion was recorded when *O*. *maius* was in direct contact with the plant roots, as compared with the indirect interaction, while no significant differences were recorded for the root biomass (Fig 1d). Interestingly, the increase in root biomass induced by *O*. *maius* correlated with a pronounced shortening of the primary root and an increase of lateral root (LR) length and number, leading to a particular clumped root phenotype well visible in Fig 1a.

### An *O*. *maius* GOGAT mutant could not induce the *A*. *thaliana* clumped root phenotype

**Fig 2:**
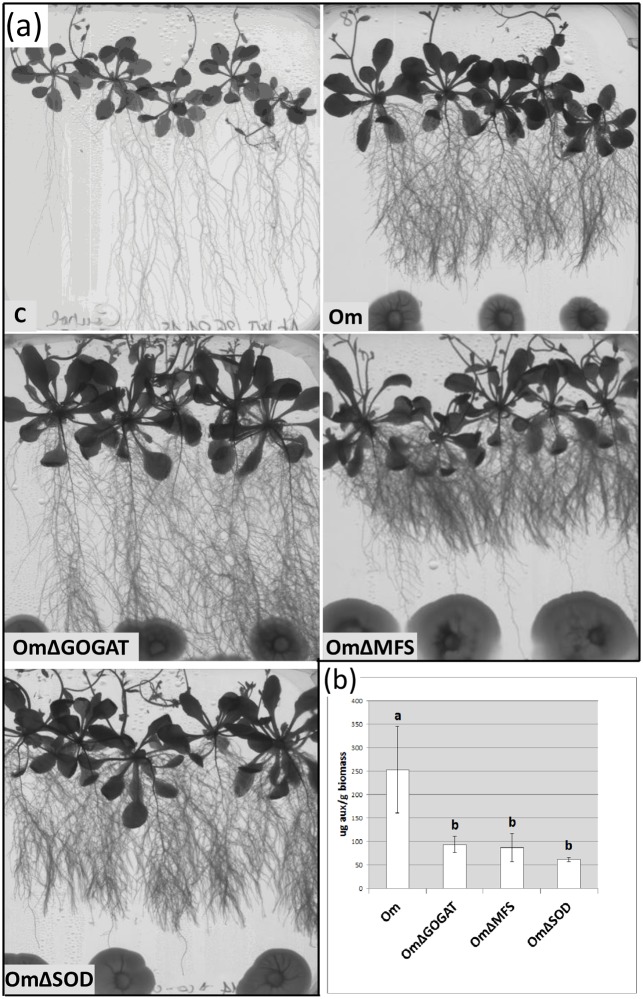
*A*. *thaliana* development in the presence of *O*. *maius* WT (Om) and of three *O*. *maius* mutants (OmΔGOGAT; OmΔMFS; OmΔSOD). (a) Control plants (C) and plant-fungus co-cultures 30 days after inoculation (all pictures were taken at the same magnification) (b) Measurement of auxin quantity released in the culture medium by *O*. *maius* WT and by the three *O*. *maius* mutants, using the Salkowski reaction [46]. Auxin quantity measured was normalized to the mycelium biomass. Bars represent the mean ±SD, n = 3 (each biological replicate represents the total biomass of 5 *A*. *thaliana* seedlings grown in an individual plate). Statistically significant differences (P<0.05) among treatments are indicated by different letters above the bars.

The use of genetic mutants is a powerful tool to associate a particular phenotype to specific genes. Therefore, three available characterized *O*. *maius* mutants (see “Fungal strains and culture media” paragraph) were also tested: two of them induced in *A*. *thaliana* the same clumped root phenotype as the wild-type strain, whereas the *O*. *maius* GOGAT mutant, altered in N metabolism, did not (Fig 2a).

### Morphometric analysis of *A*. *thaliana* root development in the presence of the *O*. *maius* WT and of the *O*. *maius* GOGAT mutant

**Fig 3:**
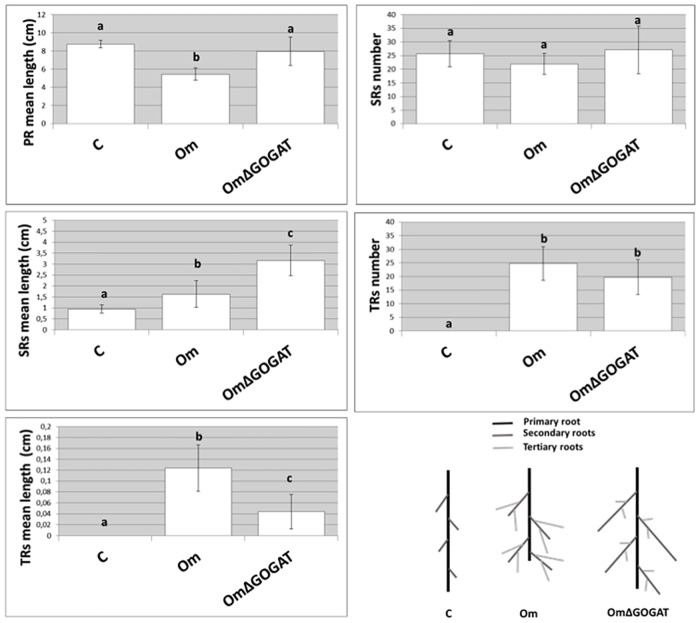
Analysis of *A*. *thaliana* root development. Average quantification of root parameters in 20 individual *A*. *thaliana* plants grown alone or co-cultured for 9 days with the *O*. *maius* WT strain (Om) or the *O*. *maius* GOGAT mutant (OmΔGOGAT) is plotted into charts. Primary (PR), secondary (SRs) and tertiary roots (TRs) were counted and measured using the ImageJ plug in SmartRoot software. A diagrammatic representation of *A*. *thaliana* root development in the different conditions tested is also shown.

To better describe the two different *A*. *thaliana* root phenotypes observed after co-cultivation with the *O*. *maius* WT and the *O*. *maius* GOGAT mutant, plant root development was followed in a time course experiment (S4 Fig) and morphometric analyses were performed after 9 days of co-culture using the SmartRoot software tool. Average values of the number and length of three different root orders -primary (PR), secondary (SRs) and tertiary roots (TRs)- were calculated for each replicate (Fig 3). When compared to control plants, the root phenotype in the presence of the *O*. *maius* WT strain was characterized by PR shortening, an increase of SR and TR length and an increase of TR number (Fig 3). On the other hand, no significant PR shortening was observed in the presence of the *O*. *maius* GOGAT mutant. For these plants, an increase of SR and TR length and an increase of TR number were measured (Fig 3). If compared to control plants, the SR length increased more in the presence of the *O*. *maius* GOGAT mutant (3.2 times) than the WT strain (1.6 times) (Fig 3). On the other hand, TRs were 2.8 times longer in the WT strain than in the mutant strain (Fig 3).

### Auxin is not involved in the *A*. *thaliana—O*. *maius* interaction

Felten et al. [29] showed that LR stimulation in *A*. *thaliana* by an ECM fungus paralleled an increase of auxin in the root apices. As alterations of nitrogen metabolism could affect auxin production [48], we tested whether the inability of the *O*. *maius* GOGAT mutant to induce the clumped root phenotype in *A*. *thaliana* could be related to a different production of fungal auxin. The amount of IAA produced by the wild type *O*. *maius* strain was significantly higher when compared to the amount produced by the three mutants tested in the plate assay (Fig 2b). However, since auxin production by the *O*. *maius* GOGAT mutant was similar to the other two mutants, this result would exclude a possible role of fungal auxin in the *A*. *thaliana* clumped root phenotype. We also verified possible induction of auxin accumulation in the plant tissues by using the *A*. *thaliana* DR5::GUS [49], that carries the promoter of the auxin-responsive DR5 gene fused with the GUS reporter gene, co-cultivated with *O*. *maius* WT and with the *O*. *maius* GOGAT mutant. After GUS staining, the blue dye was localized in the root apex and in the vascular tissues of the primary root (S1 Fig) as well as in the LR primordia, and in the secondary root tips (S1 Fig). Auxin was also detected in some areas of the leaf margin (S1 Fig). However, no differences were observed in the distribution and accumulation of GUS staining in *A*. *thaliana* roots and leaves, either in the absence or in the presence of the *O*. *maius* WT and the *O*. *maius* GOGAT mutant (S1 Fig). All together, these results suggest that auxin is probably not involved in the induction of the clumped root phenotype or in plant growth promotion by *O*. *maius*.

### VOCs do not seem responsible for increased *A*. *thaliana* biomass

**Fig 4:**
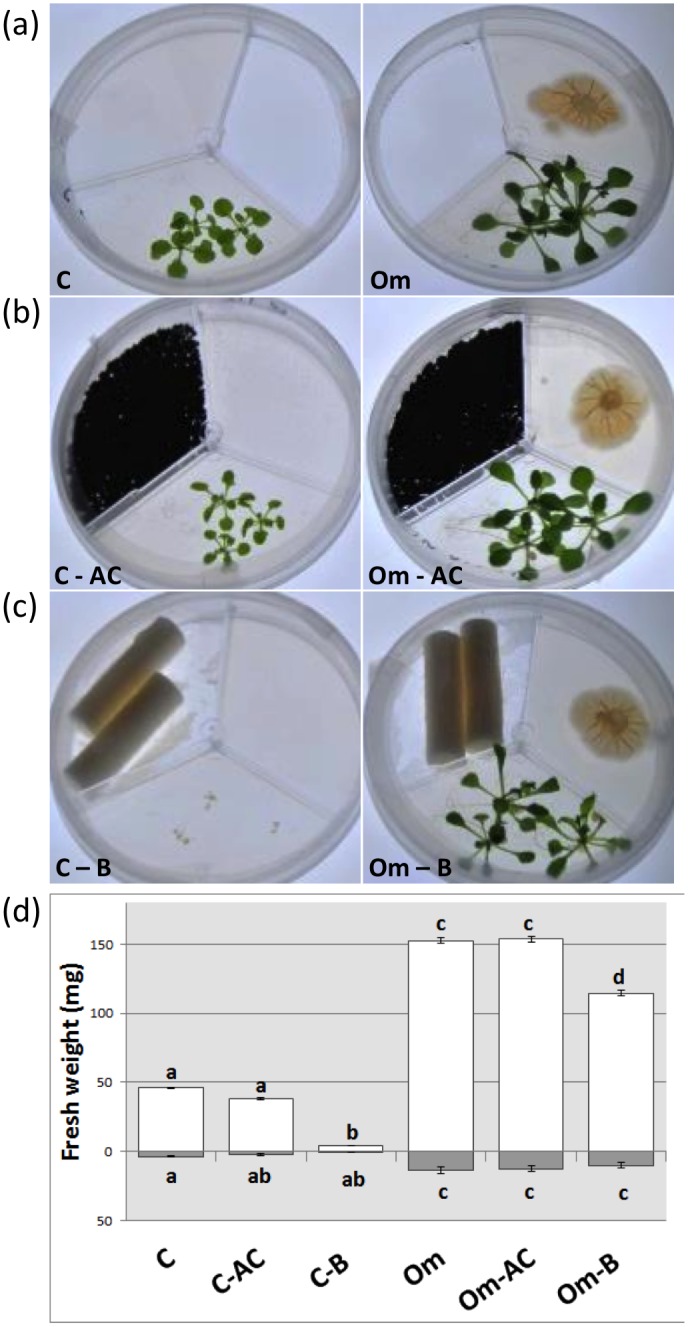
*O*. *maius—A*. *thaliana* co-cultivation experiments in the tripartite plate system. (a) Control plants and plant-fungus co-culture 15 days after inoculation; (b) same as in (a) but plates were added with a VOC trap (activated charcoal, AC) in the third compartment; (c) same as in (a) but plates were added with a CO_2_ trap [Ba(OH)_2*_8H_2_O, B] together with two dental rolls in the third compartment; (d) plant biomass measurements (roots—grey bars—and aboveground portions—open bars) in the presence/absence of the fungus and of the trap compounds. Note the strong plant biomass increase in the presence of *O*. *maius* in all the conditions tested. Bars represent the mean ±SD, n = 5 (each biological replicate represents the total biomass of 3 *A*. *thaliana* seedlings grown in an individual plate). Statistically significant differences (P<0.05) among treatments are indicated by different letters above the bars.

As previous papers reported a role of fungal-derived VOCs in root development induced by fungi [32], further experiments were performed to investigate a potential role of *O*. *maius* volatiles in *A*. *thaliana* growth promotion. Co-cultivation experiments were set up in tri-partite plates that only allowed air contact between *A*. *thaliana* and *O*. *maius* (Fig 4a). Plates containing a VOCs trap (activated charcoal) and a CO_2_ trap (barium hydroxide) were also used in parallel (Fig 4b and 4c). As the *O*. *maius* GOGAT mutant behaved differently from the *O*. *maius* WT in the square non-compartmented plates, this mutant strain was also tested in the tri-partite plate setup (S2 Fig). After 15 days of co-cultivation, *A*. *thaliana* roots and aerial portions were collected and fresh and dry weights were recorded (Figs 4d and S2d). Growth of *O*. *maius* mycelia was also recorded, and was similar in the different conditions tested (data not shown). When *A*. *thaliana* was grown in the absence of the fungus, no significant differences were measured between control plates and those added with activated charcoal. By contrast, *A*. *thaliana* biomass was significantly lower on plates containing the CO_2_ trap barium hydroxide (Figs 4c and 4d, S2c and S2d). To exclude possible phytotoxic effects of barium hydroxide, *A*. *thaliana* was grown in plates containing a CO_2_-saturated barium hydroxide solution. In this experiment, plant growth was not significantly different from control plates without barium hydroxide (S3 Fig). These results excluded a phytotoxic effect of barium hydroxide and confirmed that the dwarf plant phenotype was caused by CO_2_ depletion.

**Fig 5:**
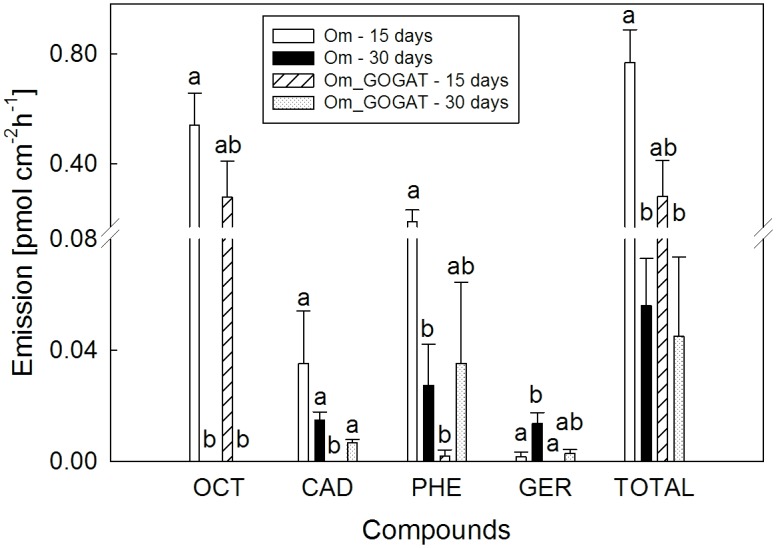
VOC emission profiles of the *O*. *maius* WT and of the *O*. *maius* GOGAT mutant. VOCs were collected in the headspace of culture plates 15 (open and hatched bars) and 30 (black and dotted bars) days after inoculation. Bars represent the mean ±SD as pmol cm^-2^ h^-1^, n = 6. (OCT) 1-octen-3-ol; (CAD) epsilon-cadinene; (PHE) phenol,2,4-bis(1,1-dimethylethyl); (GER) germacrene D.

Significant growth induction of both roots and aerial parts of *A*. *thaliana* was recorded in all treatments where the fungus, either *O*. *maius* WT or the GOGAT mutant, was present, indicating a release of growth-promoting volatile molecules. However, a similar *A*. *thaliana* biomass was recorded in the absence and in the presence of activated charcoal, an effective VOCs trap [50]. This result suggested that growth-promoting compounds were likely not VOCs (Figs 4b and 4d, S2b and S2d). Similar conclusion derived from direct measurement of VOCs emission by *O*. *maius* WT and GOGAT mutant. The results revealed in general a very low release of VOCs from both fungal strains (Fig 5). The typical fungal odor compound, 1-octen-3-ol, was emitted exclusively by 15 days old fungi, whereas emission of the sesquiterpene germacrene D was higher in the older fungal culture (Fig 5). Some of the compounds, such as the fungicide phenol,2,4-bis(1,1-dimethylethyl), showed higher emission for *O*. *maius* WT than for the *O*. *maius* GOGAT mutant (Fig 5), but it might be due to the smaller mycelium size of the mutant at the time of measurement.

In the presence of barium hydroxide, both *O*. *maius* WT and GOGAT mutant rescued the dwarf *A*. *thaliana* phenotype caused in the control plates by the presence of this CO_2_ trap. Growth of the aerial plant portion was slightly less when compared to the other co-culture conditions tested (Figs 4c and 4d, S2c and S2d), likely because part of the CO_2_ emitted by the fungus was adsorbed by, and saturated, the barium hydroxide trap.

### Various fungal strains promoted *A*. *thaliana* growth in non-compartmented and compartmented plates

**Fig 6:**
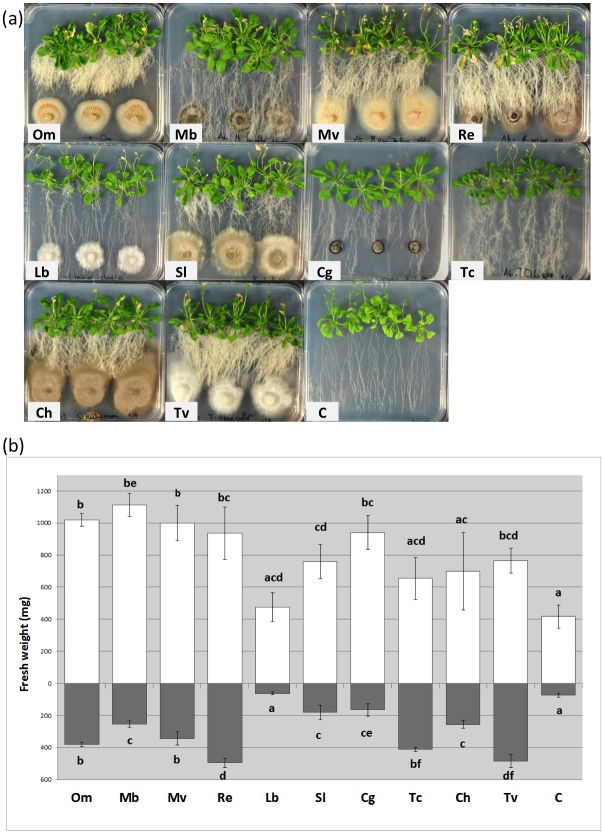
*A*. *thaliana* development in the presence of *O*. *maius* and of nine other fungi. (a) Control plants (C) and plant-fungus co-cultures 30 days after inoculation; (b) plant biomass measurements (roots—grey bars—and aboveground portions—open bars) in the presence/absence of fungi. Note the strong plant biomass increase in the presence of some of the fungi tested. Bars represent the mean ±SD, n = 5 (each biological replicate represents the total biomass of 5 *A*. *thaliana* seedlings grown in an individual plate). Statistically significant differences (P<0.05) among treatments are indicated by different letters above the bars. Om, *Oidiodendron maius*; Mb, *Meliniomyces bicolor*; Mv, *Meliniomyces variabilis*; Re, *Rhizoscyphus ericae*; Lb, *Laccaria bicolor*; Sl, *Suillus luteus*; Cg, *Cenococcum geophilum*; Tc, *Tulasnella calospora*; Ch, *Cladosporium herbarum*; Tv, *Trametes versicolor*.

**Fig 7:**
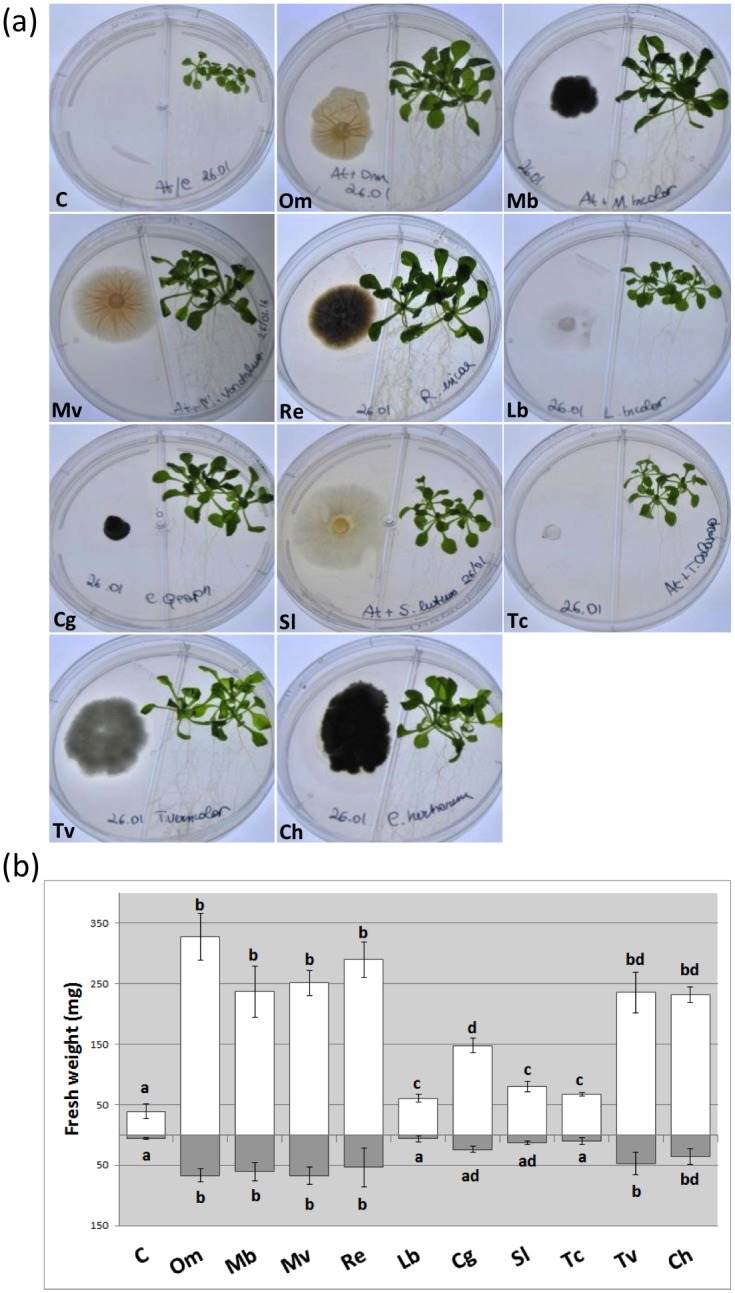
*A*. *thaliana* development in the presence of different fungi in the bipartite plate system. (a) Control plants and plant-fungus co-cultures 15 days after inoculation; (b) plant biomass measurements (roots—grey bars—and aboveground portions—open bars) in the presence/absence of fungi. Note the strong plant biomass increase in the presence of some of the fungi tested. Bars represent the mean ±SD, n = 5 (each biological replicate represents the total biomass of 3 *A*. *thaliana* seedlings grown in an individual plate). Statistically significant differences (P<0.05) among treatments are indicated by different letters above the bars. Om, *Oidiodendron maius*; Mb, *Meliniomyces bicolor*; Mv, *Meliniomyces variabilis*; Re, *Rhizoscyphus ericae*; Lb, *Laccaria bicolor*; Cg, *Cenococcum geophilum*; Sl, *Suillus luteus*; Tc, *Tulasnella calospora*; Tv, *Trametes versicolor*; Ch, *Cladosporium herbarum*.

To investigate whether the clumped root phenotype and the biomass increase observed in *A*. *thaliana* were specifically induced by *O*. *maius*, different fungal strains were tested using the non-compartmented (Fig 6) and compartmented (Fig 7) plate setups. Among the fungal strains tested, two other ERM fungi (*M*. *variabilis* and *R*. *ericae*), the saprotrophic fungus *T*. *versicolor* and the pathogenic fungus *C*. *herbarum* induced the clumped root phenotype, while the ERM fungus *M*. *bicolor*, two ECM fungi (*L*. *bicolor* and *C*. *geophilum*) and the orchid mycorrhizal fungus *T*. *calospora* did not (Fig 6a). An intermediate situation was observed for *S*. *luteus* (Fig 6a). Irrespective of the root phenotype, most of these fungi significantly increased the biomass of both *A*. *thaliana* roots and aerial portions (Fig 6b), in particular the four ERM fungi, the ECM fungi *S*. *luteus* and *C*. *geophylum* and the saprotrophic fungus *T*. *versicolor* (Fig 6b). It should be also noted that, irrespective of the biomass increase and root phenotype, some other developmental traits of *A*. *thaliana* (e.g. flowering, leaf area) were depending on the fungus in the co-cultivation plates, despite the fact that seedlings were all at the same developmental stage at the beginning of the experiment (Fig 5b).

The same fungi tested in the non-compartmented square plates were also tested in bipartite plates, which allow only volatile molecules to be exchanged between the two partners (Fig 7). Similarly to *O*. *maius* WT, no clumped root phenotype was observed in this experimental setup for any of the fungi tested (Fig 7a), but a significant increase in plant biomass was recorded for most of them after 15 days of co-cultivation (though fungal growth promotion started to be visible after 7 days of co-cultivation), although to a different extent (Fig 7b). The most pronounced plant growth was induced by the four ericoid strains, by *T*. *versicolor* and by *C*. *herbarum*, whereas the three ECM fungi and the orchid mycorrhizal fungus were less effective in promoting plant growth (Fig 7b). These fungi were also those producing the lowest biomass in our experimental conditions (S1 Table). Pearson’s correlation test showed, with very few exceptions, a significant correlation (P<0.01) between plant and fungal biomass (S1 Table). As expected, a positive correlation was found between fungi producing a large biomass and the increase of above- and belowground plant portions, regardless of their ecological strategies. This experiment strengthened therefore the hypothesis that fungi induce plant growth in our *in vitro* experimental setup due to a nonspecific mechanism (i.e. CO_2_ emission).

## Discussion

### *O*. *maius* deeply influences development of a non-host plant

ERM fungi have been frequently found to associate with plant species outside the Ericaceae (see [36] and references therein), where they may form structures typical of fungal endophytes [39], but the role of these fungi when interacting with non-ericaceous plants is unclear. Here, we showed that *O*. *maius* significantly promotes growth of both aerial and root portions of *A*. *thaliana in vitro*. In addition, a peculiar clumped root phenotype was induced by this ericoid mycorrhizal fungus as a result of increased lateral root length and number associated to shortening of the primary root growth. Other fungi tested for comparison also promoted *A*. *thaliana* growth and influenced plant development, with some of them causing the peculiar clumped root phenotype, a behavior that may be quite widespread among soil fungi (this work; [19]). The ability to increase plant growth and to modify plant development and root architecture is largely documented for both PGPRs and PGPFs. The rhizobacterium *Pseudomonas aeruginosa* modifies root architecture in *A*. *thaliana* by increasing primary root length, number of lateral roots (LR) and root fresh weight [12] while *Serratia marcescens*, when co-cultured with *A*. *thaliana*, inhibited primary root elongation and induced LR in a distance-dependent manner [11]. *Azospirillum brasilense* inhibited root length while enhancing root hair formation in an inoculum concentration-dependent manner in *Triticum aestivum* [8]. Sirrenberg et al. [15] showed that the endophytic fungus *Piriformospora indica* increased root growth and branching in *A*. *thaliana* and Rai and Varma [51] showed that the same fungus increased root proliferation in *Adhatoda visica*. Enhanced *A*. *thaliana* root development and branching was reported also for three endophytic fungi isolated from *Mentha aquatica* [19]. Two free-living fungi, *Trichoderma virens* and *Trichoderma atroviride*, enhanced LR growth when co-cultured with *A*. *thaliana* [17]. Alteration of root morphology by mycorrhizal fungi is also well known, both in host and non-host plants. In *Oryza sativa* and *Zea mays*, fine and large lateral root formation is stimulated by *Rhizophagus irregularis* [23,52], while *R*. *clarus* and *Gigaspora decipiens* enhance root biomass in *Aquilaria filaria* and *Dyera polyphylla* [22]. ECM fungi also modified primary root growth and increased LR formation in plants like *Populus*, *Picea abies*, *Pinus* spp. and *Cistus incanus*, and they also modified root development and branching of the non-host *A*. *thaliana* [25–29,31]. Heller et al. [30] reported that LR production in *Pinus sylvestris* inoculated with *Laccaria bicolor* was faster than in non-inoculated plants. All these alterations of root architecture, due to interactions with soil microorganisms, could represent an advantage for plants by improving nutrient acquisition and tolerance to abiotic and biotic stress [53,54]. Ericoid mycorrhizal (ERM) fungi modify root development of their host plant and Villareal-Ruiz et al. [55] showed significant effects of a fungus of the *Rhizoscyphus ericae* aggregate on *Vaccinium* root development. The most profound effects were on total hair root length (eight-fold increase in the presence of the fungus) and the number of hair root tips (six-fold increase in the presence of the fungus). By contrast, the effect of ERM fungi on growth and development of non-host plants has never been investigated, to our knowledge.

### Which molecules are involved in the interaction between *O*. *maius* and *A*. *thaliana*?

The results derived from plant-fungus co-cultures in compartmented and non-compartmented plates suggest that the two main phenotypes observed in *A*. *thaliana*, i.e. the increase in plant biomass and the clumped root phenotype, are induced by different compounds.

Several molecules produced by PGPFs have been described as being involved in plant growth promotion, such as cytokinins and gibberellins, brassinosteroids, oligosaccharines, bioamines, salicylic acid, and jasmonic acid [13,17,56]. In addition, PGPFs can produce molecules affecting hormone homeostasis in plants [13,14,56]. Some microorganisms, including ECM fungi, recruit the auxin signaling pathway to change plant root architecture [29,31], and LR induction has been specifically attributed to redistribution of auxin transporters at the root apex [29]. The inhibition of primary root growth by *Trichoderma* spp. has been attributed to an increase of the auxin content and to a disruption of the auxin response gradients in root tips induced by peptaibols, a class of linear peptide antibiotics [57].

For ERM fungi, Berta and Gianinazzi-Pearson [58] suggested either fungal auxin production or stimulation of plant hormone production as a possible cause of the substantial change in root length and number of hair roots in *Calluna vulgaris* seedlings infected with *R*. *ericae*.

Auxin was identified in culture filtrates of *R*. *ericae* strains [59], and here we also showed auxin production by *O*. *maius*. This aspect will be further discussed in the following section, but our experiments with *A*. *thaliana* DR5::GUS suggest that the plant phenotype in the presence of *O*. *maius* cannot be ascribed to accumulation of this plant hormone.

Volatile compounds can also influence root development. Splivallo et al. [31] mentioned the influence of ethylene, a volatile hormone, on *A*. *thaliana* LR development caused by the ECM fungi *Tuber borchii* and *T*. *melanosporum*. Ditengou et al. [32] demonstrated that LR branching induced by *L*. *bicolor* in *A*. *thaliana* could be ascribed to a specific VOC, a sesquiterpene. Garnica-Vergara et al. [60] showed that the 6-pentyl-2H-pyran-2-one (6-PP), a major VOC biosynthesized by *Trichoderma* spp., promoted plant growth and regulated root architecture by modulating the expression of auxin-transport proteins. Sánchez-López et al. [61] suggested that VOCs emitted by different rhizospheric and non-rhizospheric bacteria and fungi enhanced plant growth, photosynthesis efficiency, cytokinin levels, sugars accumulation and flowering. We tested whether the increase in plant biomass and the induction of the clumped root phenotype may be due to soluble or volatile compounds by using compartmented plates, where only volatile compounds emitted by *O*. *maius* could be perceived by *A*. *thaliana*. Whereas a significant increase in plant biomass was still observed in the compartmented plates, the peculiar clumped root phenotype was only induced when *O*. *maius* and *A*. *thaliana* were co-cultured in non-compartmented plates, suggesting a soluble diffusible signal. The experiments in compartmented plates in the presence and absence of activated charcoal, a VOCs trap [50], also suggested that VOCs likely play no role in the plant growth promotion induced by *O*. *maius*. This conclusion is supported by the fact that VOCs emission by *O*. *maius* was low in the growth conditions and at the developmental stage chosen for measurements. Although it cannot be excluded that *O*. *maius* VOCs emission could change under different environmental or physiological conditions, similarly to what was recently shown for *Alternaria* and *Fusarium* spp. [34], it should be noted that only two genes putatively coding for terpene synthases were found in the *O*. *maius* genome. By contrast, eight putative terpene synthase genes were found in *L*. *bicolor* [32].

Similarly to *O*. *maius*, a general increase in *A*. *thaliana* biomass was observed in the bipartite plates, where only volatile molecules could reach the plant, with all other fungi tested, whereas the clumped root phenotype was only observed for some fungi, including all ERM fungi but *M*. *bicolor*, in the non-compartmented petri plates, where soluble molecules could diffuse from the fungus to the plant.

The results with the compartmented plates in the presence of CO_2_ traps support the involvement of fungal produced CO_2_ in plant growth promotion, at least in our experimental setup, either through increased photosynthetic carbon fixation or as a developmental signal. Increased root biomass may in fact indirectly derive from higher carbon translocation from the photosynthetic leaves, but carbon dioxide is also known to stimulate primary root elongation and root branching [62,31]. Similarly, Kai and Piechulla [63] suggested that *A*. *thaliana* growth promotion by the rhizobacterium *Serratia odorifera* was due to the microbial CO_2_ accumulation in the co-cultivation plates. Thus, fungal derived CO_2_ is likely responsible for the increased plant biomass observed in the bipartite plates, whereas an as yet unidentified soluble diffusing molecule must be responsible for the clumped root phenotype, characterized by primary root shortening and increased LR length and number.

### The *O*. *maius* GOGAT mutant suggests possible relationships between nitrogen metabolism and the clumped root phenotype in *A*. *thaliana*

Previous authors have reported a clumped root phenotype in *A*. *thaliana* in the presence of both bacteria and fungi [6,11,19,31]. Although auxin can induce primary root growth inhibition and has been suggested to be responsible for this particular phenotype in *A*. *thaliana* [31], our results with the *O*. *maius* GOGAT mutant seem to exclude the involvement of fungal-derived auxin. The *O*. *maius* GOGAT mutant, recently characterized by Khouja et al. [43], carries a partial deletion of the glutamate synthase (NADH-GOGAT EC 1.4.1.14) gene. Glutamate (Glu) biosynthesis in this mutant is therefore only mediated by the NADP-dependent glutamate dehydrogenase (NADP-GDH, EC 1.4.1.4), in contrast with the WT strain, where two different metabolic pathways (GOGAT and GDH) for Glu biosynthesis are working [43]. Glu has been reported to be a signalling molecule in roots [64–67] and it is the amino acid whose effects on root development in several plant species are most distinctive [64–67]. In *A*. *thaliana*, where these effects have been studied, Glu inhibited primary root growth and stimulated the outgrowth of lateral roots, producing a shorter and more branched root system [68]. We obtained, in our experimental setup, the same Glu effect on *A*. *thaliana* seedlings, with shorter and more branched root (S5 Fig). Interestingly, this is the phenotype we observed in *A*. *thaliana* in the presence of the *O*. *maius* WT strain, and it is tempting to speculate that the *O*. *maius* GOGAT mutant may be unable to induce the clumped root phenotype in *A*. *thaliana* because, due to the *OmGOGAT* deletion, it produces and releases into the medium a lower amount of glutamate. Glutamate and glutamine are also very important for maintenance and promotion of cell function because they are substrates for protein synthesis, regulate acid-base balance, provide nitrogen transport, and act as precursors of nucleotide and nucleic acid synthesis and of glutathione production [69]. It is therefore highly possible that the GOGAT mutation, by modifying Glu synthesis, may indirectly affect the biosynthesis of other important molecules, which in turn could be involved in the modification of root architecture. Further investigations of the metabolites differentially released by *O*. *maius* WT and the GOGAT mutant would help to verify changes in Glu secretion, or to identify other potential diffusible signals capable of inducing this peculiar clumped root phenotype in *A*. *thaliana*.

Another possible explanation for the different root growth pattern observed in the presence of the *O*. *maius* WT and the *O*. *maius* GOGAT mutant could be related to their different exploitation of nitrogen sources in the growing medium. Both nitrate and ammonium were available in the growth substrate, but they could be differently accessed by the WT and the mutant strains due to their different nitrogen pathways. The inhibitory effect of ammonium on primary root growth has been well documented [70], as well as the stimulation of LR growth by nitrate [65]. A different exploitation rate of the nitrogen sources available in the culture media by the *O*. *maius* WT and by the *O*. *maius* GOGAT mutant could lead to a different concentration of these two nitrogen sources in the co-culture plates and therefore to a different plant root phenotype.

## Conclusions

*A*. *thaliana* is becoming a recognized model to analyse both mutualistic and non-mutualistic plant-microbe interactions [71–74], and several PGPFs have been shown to promote growth of this plant *in vitro* [6,17,19]. We have confirmed this observation for ERM fungi, as well as for other mycorrhizal and non-mycorrhizal fungi. However, it seems that the general increase in plant biomass was mainly caused, in our experimental setup, by carbon dioxide produced by the fungal mycelium and accumulated in the *in vitro* conditions, rather than by specific VOC signals. This finding should raise awareness for the interpretation of the results of plant-fungus co-cultivation experiments. Indeed a very recent review [50] pointed out that microbial CO_2_ in sealed co-culture plates may lead to a general increase of plant growth similar to what can be observed in the presence of microbial volatiles, suggesting that care should be taken when ecologically relevant functions of microbial VOCs are studied. On the other hand, from an ecological point of view, soil respiration is a key ecosystem process that releases carbon from the soil organic matter, thus playing an important role in global carbon cycling [75–76]. Carbon dioxide released through respiration by fungi closely associated with plants can be used by plants covering the soil surface for photosynthesis, leading to healthier plants, with an increased nutrient uptake capacity due to a more developed root system and with a higher aboveground biomass.

In addition to the general biomass increase due to fungal respiration, *O*. *maius* as well as about half of the other fungi tested induced in *A*. *thaliana* a peculiar clumped root phenotype, likely caused by diffusible soluble fungal compounds. Although the nature of these fungal compounds is as yet unknown, the inability of the *O*. *maius* GOGAT mutant to induce this root phenotype should help us to elucidate the mechanisms involved.

## Supporting Information

S1 Fig GUS assay results for the *A*. *thaliana* DR5::GUS line plants. (a) *A*. *thaliana* DR5::GUS control plants, (b) *A*. *thaliana* DR5::GUS plants co-cultivated with the *O*. *maius* WT (b) and with the *O*. *maius* GOGAT mutant (c). Staining was performed on aboveground and belowground portions of *A*. *thaliana* fresh tissues and stained tissues were observed and photographed using a Nikon Eclipse E400 optical microscope. The staining was observed in the root apex, in the vascular tissues of the primary root, in the lateral root primordia, and in some areas of the leaf margin. No differences for dye distribution and accumulation in plant tissues were observed in the absence or in the presence of fungi. Bars = 100 μm.
(TIF)

S2 Fig *O*. *maius* GOGAT mutant—*A*. *thaliana* co-cultivation experiments in the tripartite plate system. (a) Control plants and plant-fungus co-cultures 15 days after inoculation; (b) same as in (a) but plates were added with a VOCs trap compound (activated charcoal, AC) in the third compartment; (c) same as in (a) but plates were added with a CO_2_ trap compound [Ba(OH)_2*_8H_2_O, B] in the third compartment; (d) plant biomass measurements (roots—grey bars—and aboveground portions—open bars) in the presence/absence of the fungus and of the trap compounds. Note the strong plant biomass increase in the presence of the *O*. *maius* GOGAT mutant in all the conditions tested. Bars represent the mean ±SD, n = 5. Statistically significant differences (P<0.05) among treatments are indicated by different letters above the bars.
(TIF)

S3 Fig *A*. *thaliana* plants growth in the tripartite plate system with a CO_2_ saturated trap compound. (a) *A*. *thaliana* plants growth in control plates (At) and in plates added with a CO_2_ trap compound, Ba(OH)_2*_8H_2_O (At-B) and with the same compound saturated with CO_2_ (At-B-CO_2_ saturated); (b) plant biomass measurements (roots—grey bars—and aboveground portions—open bars) in the presence/absence of the CO_2_ trap compound saturated or not with CO_2_. The saturation with CO_2_ of the barium hydroxide solution rescued the plant phenotype observed in the absence of CO_2_ trap compounds. Bars represent the mean ±SD, n = 5. Statistically significant differences (P<0.05) among treatments are indicated by different letters above the bars.
(TIF)

S4 Fig Time course of the *A*. *thaliana* plants development in the absence/presence of the *O*. *maius* WT and of the *O*. *maius* GOGAT mutant. The clumped root phenotype started forming after 6 days of plant-fungus co-cultivation only in the presence of the *O*. *maius* WT strain.
(TIF)

S5 Fig *A*. *thaliana* plants development in the absence/presence of glutamate. Five days old *A*. *thaliana* plants grown for 4 days on the MS medium (C) and on the MS medium added with 25.6 mM Na-glutamate (Glu) using the non-compartmented square plate setup. Note the shorter and more branched root in the presence of glutamate.
(TIF)

S1 Table Plant and fungal biomass in the bipartite plates. Plant and fungal biomasses were measured in the bipartite plates and a correlation analysis was performed using the Pearson’s correlation test.
(DOCX)
