# Supplementary figures and images for: Ecologically Different Fungi Affect Arabidopsis Development: Contribution of Soluble and Volatile Compounds
Source: PLoS One. 2016 Dec 14;11(12):e0168236. doi: 10.1371/journal.pone.0168236 (PMC5156394; doi:10.1371/journal.pone.0168236)

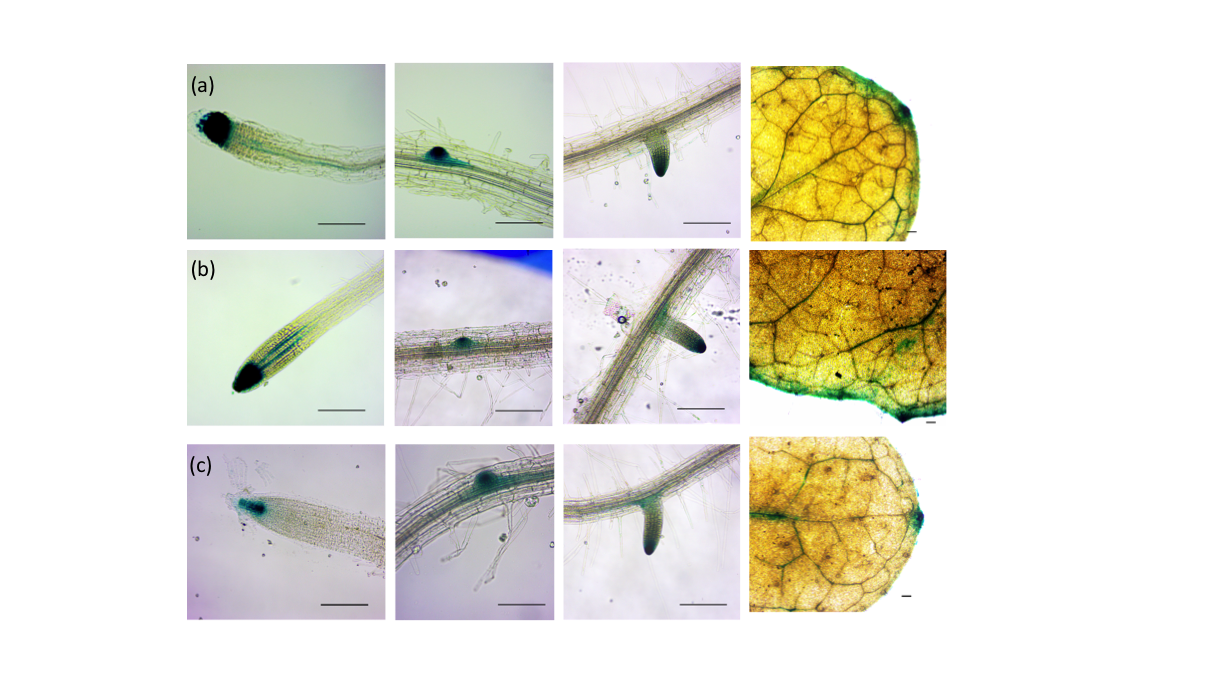

Supplement: S1 Fig — (a) A. thaliana DR5::GUS control plants, (b) A. thaliana DR5::GUS plants co-cultivated with the O. maius WT (b) and with the O. maius GOGAT mutant (c). Staining was performed on aboveground and belowground portions of A. thaliana fresh tissues and stained tissues were observed and photographed using a Nikon Eclipse E400 optical microscope. The staining was observed in the root apex, in the vascular tissues of the primary root, in the lateral root primordia, and in some areas of the leaf margin. No differences for dye distribution and accumulation in plant tissues were observed in the absence or in the presence of fungi. Bars = 100 μm. (TIF) [file pone.0168236.s001.tif]

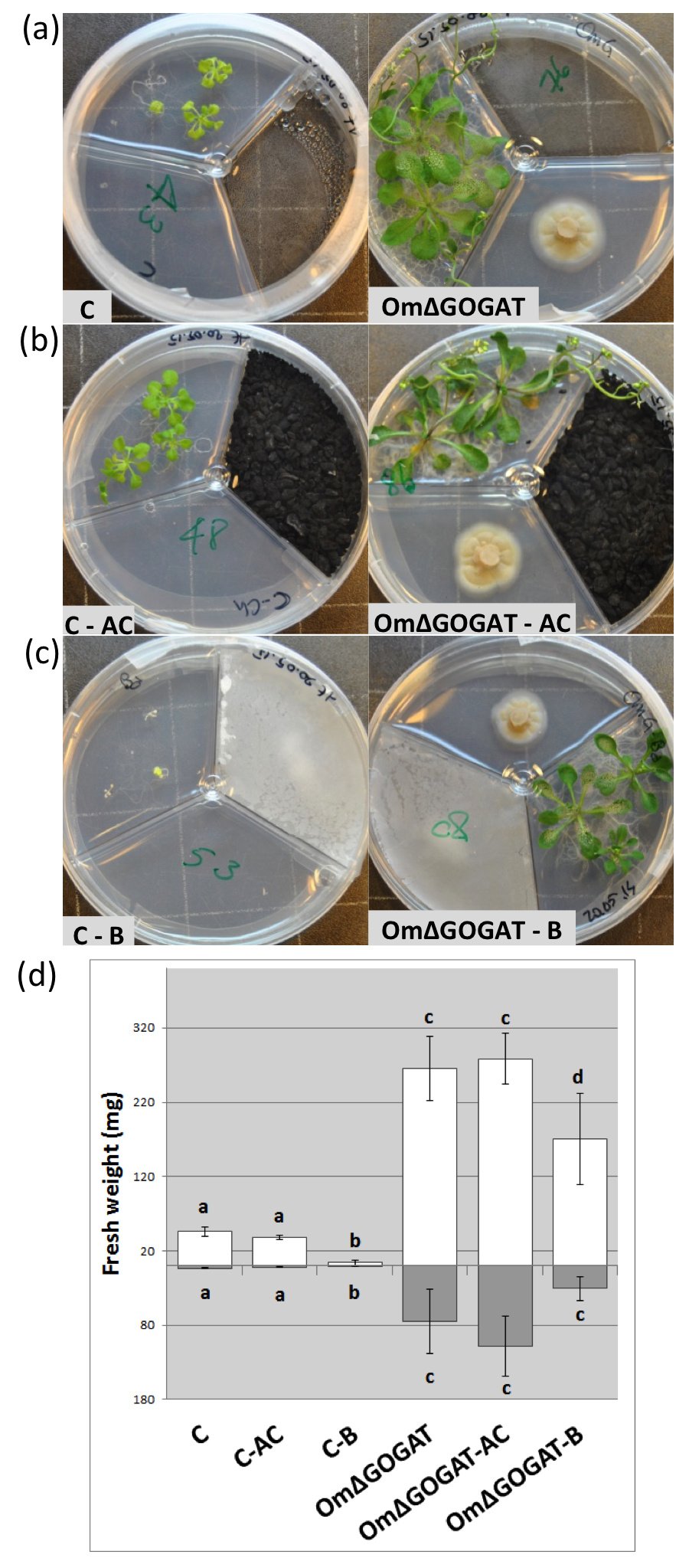

Supplement: S2 Fig — (a) Control plants and plant-fungus co-cultures 15 days after inoculation; (b) same as in (a) but plates were added with a VOCs trap compound (activated charcoal, AC) in the third compartment; (c) same as in (a) but plates were added with a CO2 trap compound [Ba(OH)2*8H2O, B] in the third compartment; (d) plant biomass measurements (roots—grey bars—and aboveground portions—open bars) in the presence/absence of the fungus and of the trap compounds. Note the strong plant biomass increase in the presence of the O. maius GOGAT mutant in all the conditions tested. Bars represent the mean ±SD, n = 5. Statistically significant differences (P<0.05) among treatments are indicated by different letters above the bars. (TIF) [file pone.0168236.s002.tif]

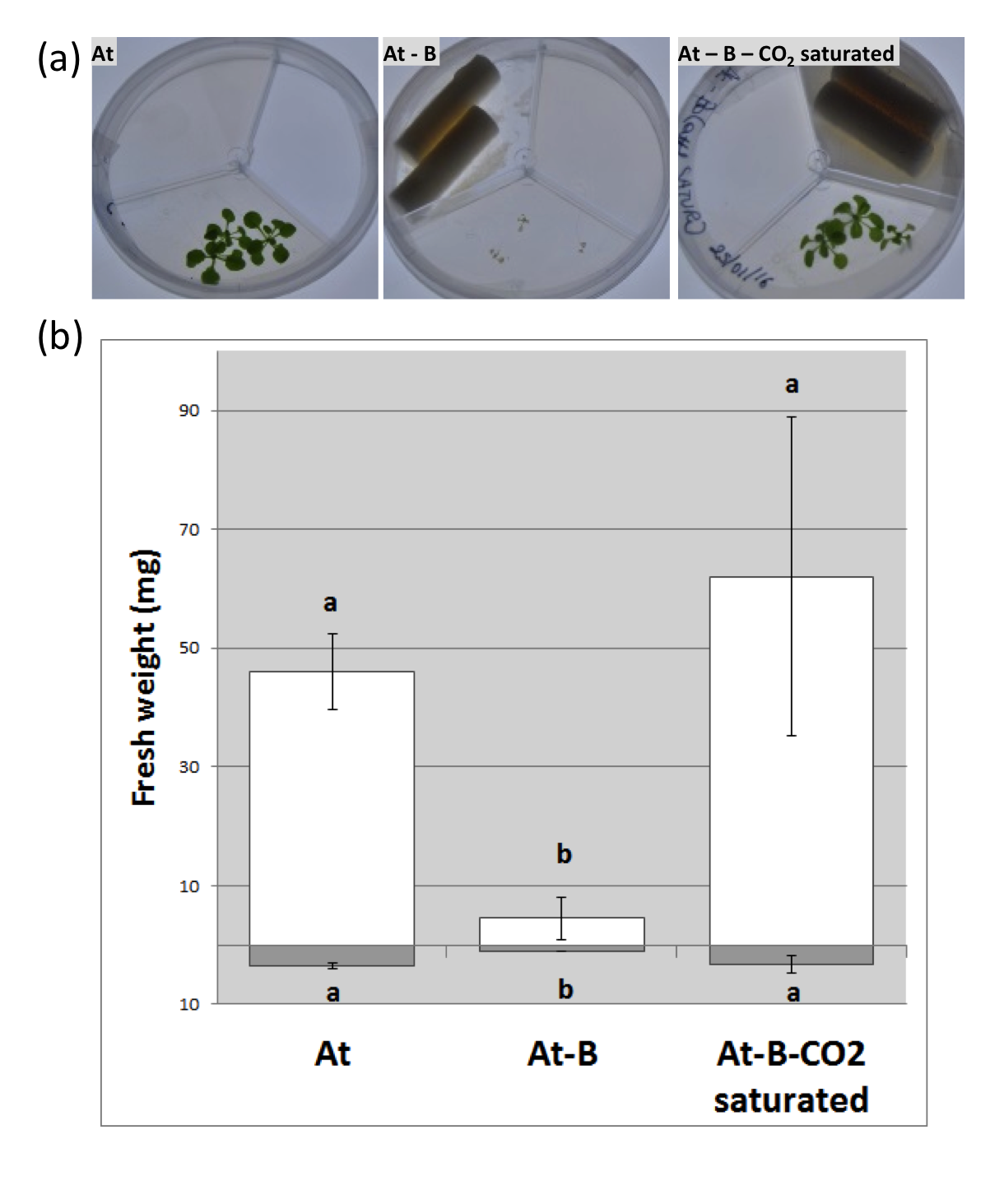

Supplement: S3 Fig — (a) A. thaliana plants growth in control plates (At) and in plates added with a CO2 trap compound, Ba(OH)2*8H2O (At-B) and with the same compound saturated with CO2 (At-B-CO2 saturated); (b) plant biomass measurements (roots—grey bars—and aboveground portions—open bars) in the presence/absence of the CO2 trap compound saturated or not with CO2. The saturation with CO2 of the barium hydroxide solution rescued the plant phenotype observed in the absence of CO2 trap compounds. Bars represent the mean ±SD, n = 5. Statistically significant differences (P<0.05) among treatments are indicated by different letters above the bars. (TIF) [file pone.0168236.s003.tif]

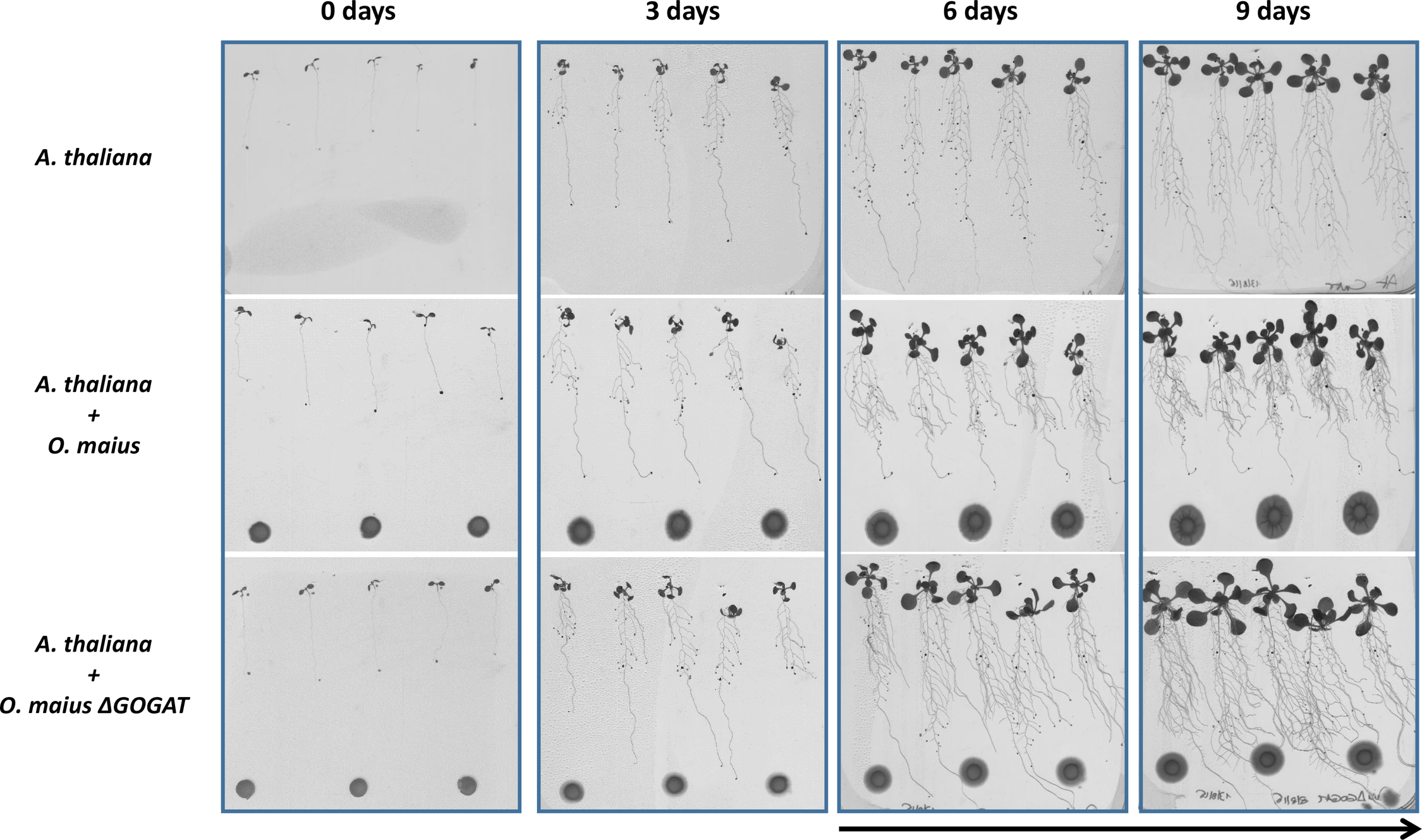

Supplement: S4 Fig — The clumped root phenotype started forming after 6 days of plant-fungus co-cultivation only in the presence of the O. maius WT strain. (TIF) [file pone.0168236.s004.tif]

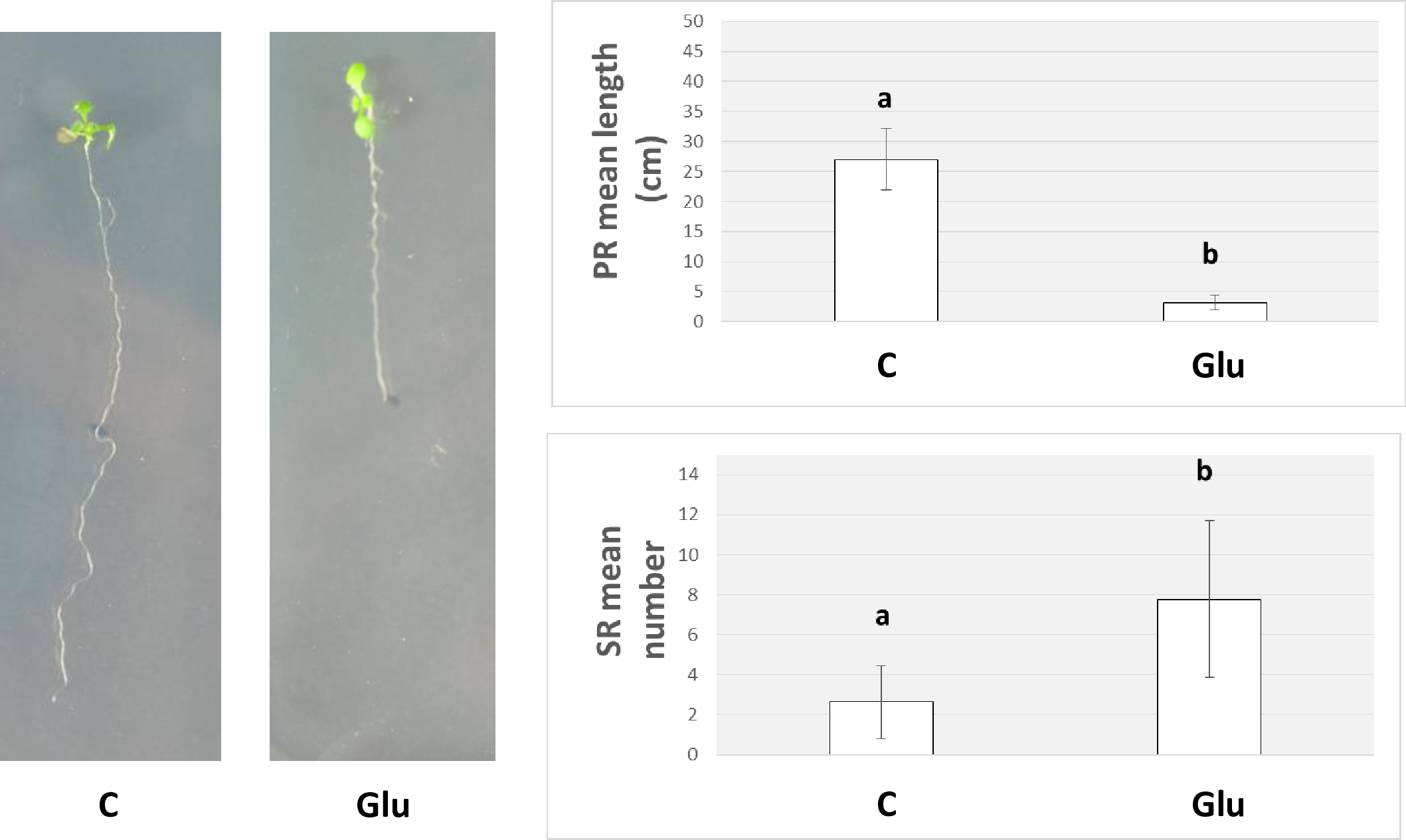

Supplement: S5 Fig — Five days old A. thaliana plants grown for 4 days on the MS medium (C) and on the MS medium added with 25.6 mM Na-glutamate (Glu) using the non-compartmented square plate setup. Note the shorter and more branched root in the presence of glutamate. (TIF) [file pone.0168236.s005.tif]
