# Supplementary material for: Ecologically Different Fungi Affect Arabidopsis Development: Contribution of Soluble and Volatile Compounds
Source: PLoS One. 2016 Dec 14;11(12):e0168236. doi: 10.1371/journal.pone.0168236 (PMC5156394; doi:10.1371/journal.pone.0168236)
Supplement: S1 Table — Plant and fungal biomasses were measured in the bipartite plates and a correlation analysis was performed using the Pearson’s correlation test. (DOCX) [file pone.0168236.s006.docx]

|  | Aboveground plant biomass (FW*) | Plant root biomass (FW*) | Mycelium biomass (FW*) | Pearson’s correlation test: aboveground plant biomass/fungal biomass | Pearson’s correlation test: root biomass/fungal biomass |
| --- | --- | --- | --- | --- | --- |
| *A. thaliana* | 39.00±12.08 | 6.20±1.79 |  |  |  |
| *A. thaliana-O. maius* | 328.00±41.58 | 67.25±14.82 | 168.75±5.12 | 0.95 | 0.99 |
| *A. thaliana-M. bicolor* | 236.60±20.61 | 60.67±14.05 | 135.75±9.71 | 0.95 | 0.99 |
| *A. thaliana-M. variabilis* | 251.20±38.53 | 67.75±11.00 | 299.00±24.00 | 0.95 | 1.00 |
| *A. thaliana-R. ericae* | 289.67±29.67 | 53.75±32.17 | 271.67±24.58 | 0.83** | 1.00 |
| *A. thaliana-L. bicolor* | 60.50±6.66 | 6.28±5.20 | 19.25±1.53 | 0.52** | 0.90 |
| *A. thaliana-C. geophilum* | 147.40±12.18 | 24.00±5.60 | 66.00±8.91 | 0.90 | 0.97 |
| *A. thaliana-S. luteus* | 80.20±8.66 | 13.25±3.30 | 16.75±2.06 | 0.83 | 0.93 |
| *A. thaliana-T. calospora* | 67.00±2.99 | 10.00±5.60 | 4.75±1.71 | 0.48** | 0.85 |
| *A. thaliana-T. versicolor* | 235.33±33.23 | 47.25±19.02 | 110.50±13.10 | 0.81 | 0.98 |
| *A. thaliana-C. herbarum* | 231.67±12.50 | 36.00±12.33 | 476.00±30.51 | 0.86 | 0.99 |
| *A. thaliana-OmΔGOGAT* | 187.80±19.70 | 53.00±4.24 | 152.23±6.15 | 0.98 | 1.00 |

**S1 Table. Plant and fungal biomass in the bipartite plates.**

Plant and fungal biomasses were measured in the bipartite plates and a correlation analysis was performed using the Pearson’s correlation test.

*FW: fresh weight (mg)

** values not significantly correlated
